# Molecular characteristics of extended-spectrum β-lactamase-producing *Escherichia coli* in Riyadh: emergence of CTX-M-15-producing E. coli ST131

**DOI:** 10.1186/1476-0711-13-4

**Published:** 2014-01-07

**Authors:** Mohamed H Al-Agamy, Atef M Shibl, Mohamed M Hafez, Mohammad N Al-Ahdal, Ziad A Memish, Harish Khubnani

**Affiliations:** 1 Pharmaceutics and Microbiology Department, College of Pharmacy, King Saud University, P.O. Box 2457, Riyadh 11451, Saudi Arabia; 2 Microbiology and Immunology Department, Faculty of Pharmacy, Al-Azhar University, Cairo, Egypt; 3 Department of Pharmacology and Toxicology, College of Pharmacy, King Saud University, P.O. Box 2457, Riyadh 11451, Saudi Arabia; 4 Cancer biology Department, Virology and Immunology Unit, National Cancer Institute, Cairo University, Cairo, Egypt; 5 Department of Infection and Immunity, King Faisal Specialist Hospital and Research Centre, Riyadh, Saudi Arabia; 6 College of Medicine, Alfaisal University, Riyadh, Saudi Arabia; 7 Preventive Medicine Directorate, Ministry of Health, Riyadh, Saudi Arabia; 8 Microbiology section, Prince Salman Hospital, Riyadh, Saudi Arabia

**Keywords:** β-lactam resistance, Class A β-lactamases, PFGE, ST131, Saudi Arabia

## Abstract

**Background:**

The prevalence of extended-spectrum β-lactamase-producing *Escherichia coli* (ESBL-EC) has increased recently. The aim of this study was to further characterise and to assess the occurrence of ESBL-EC in Riyadh, to use pulsed field gel electrophoresis (PFGE) typing to investigate the epidemiology of ESBL-EC and to determine the prevalence of ST131 in ESBL-EC.

**Methods:**

A total of 152 *E. coli* isolates were collected at a tertiary hospital in Riyadh from September 2010 to June 2011. Genotypic and phenotypic methods were used to characterise ESBLs. PFGE was used to determine genetic relatedness. Detection of ST131 and CTX-M-like ESBLs was performed using real-time PCR.

**Results:**

Of 152 strains, 31 were positive for ESBLs by phenotypic methods. The *bla*_CTX-M-15_ gene was highly prevalent (30/31 strains, 96.77%) among the 31 ESBL-positive *E. coli* strains. The *bla*_CTX-M-27_ gene was detected in one strain. Twenty (64.5%) out of 31 of ESBL-EC were ST131. PFGE revealed 29 different pulsotypes.

**Conclusions:**

Our study documented the high prevalence of ESBLs in *E. coli* isolates, with CTX-M-15 as the predominant ESBL gene. ST131 clone producing CTX-M-15 has a major presence in our hospital. The high prevalence of CTX-M producers was not due to the spread of a single clone. To the best of our knowledge, this study represents the first report of CTX-M-15 and CTX-M-27 β-lactamases and the detection of the ST131 clone in Saudi *E. coli* isolates.

## Background

Extended-spectrum β-lactamase-producing *Escherichia coli* (ESBL-EC) pose a serious threat to the successful treatment of common bacterial infections. Over the past two decades, there has been an increase in the prevalence of ESBL-EC [1]. In addition to TEM and SHV variants of ESBLs, CTX-M enzymes have replaced TEM and SHV in the past several years. CTX-M β-lactamases have emerged as the predominant ESBL type worldwide [2]. Within the CTX-M family, CTX-M-15 is currently the most widely disseminated CTX-M genotype [2,3]. Recently, several studies have highlighted the worldwide spread of the *E. coli* ST131 clone [4-6]. The successful dissemination of CTX-M-15 has been considered to be due to the dissemination of genetic elements and the clonal expansion of a pandemic *E. coli* clone, ST131, with high virulence potential [7]. The *E. coli* ST131 clone has been reported worldwide; this clone is frequently multidrug-resistant and commonly carries CTX-M-15 [6,8].

Previously published studies in Saudi Arabia have sought to determine the prevalence of ESBL-producing isolates, mainly *Klebsiella pneumoniae* and, to a lesser extent, *E. coli,* with CTX-M-15 being the most common ESBL [9-17]. However, there is a paucity of studies on the prevalence of ESBL-EC from Saudi Arabia. Until now, no detailed reports characterizing the genotypes of ESBL enzymes or the spread of the ST131 clone in ESBL-EC out of Saudi Arabia have been published. Therefore, this study was performed to investigate the molecular epidemiology and genetic characteristics of clinical ESBL-EC isolates obtained from a tertiary hospital in Riyadh, Saudi Arabia.

## Materials and methods

### Bacterial isolates

One hundred fifty-two non-consecutive, non-duplicate clinical *E. coli* isolates were obtained from inpatients hospitalised at a tertiary hospital in Riyadh, Saudi Arabia. These isolates were collected over a period of 10 months from September 2010 to June 2011. The samples contained 119 isolates from urine, 11 from sputum, 10 from stool, 9 from blood, and 3 from abscesses. Isolates were identified using traditional bacteriological methods and biochemical testing with an API 20E according to the manufacturer’s recommendations. The isolates were stored at −80°C in 15% glycerol (v/v) in tryptic soy broth.

### Antimicrobial susceptibility testing

Antimicrobial susceptibility testing was performed using the disc diffusion method of the Clinical and Laboratory Standards Institute (CLSI) with discs from BBL (Becton Dickinson, Sparks Glencoe, MD, USA). The minimum inhibitory concentration (MIC) for each antibiotic was determined using the dilution and diffusion method on Mueller–Hinton agar with E-test strips (bioMerieux, Marcy L’Etoile, France). The results were interpreted according to the current guidelines of the CLSI [18]. The following antibiotics were tested: amikacin, amoxicillin, amoxicillin–clavulanate, aztreonam, cefepime, cefotaxime, cefoxitin, ceftazidime, ceftazidime/clavulanate, ciprofloxacin, colistin, fosfomycin, gentamicin, imipenem, meropenem, piperacillin, piperacillin–tazobactam, and tigecycline. *E. coli* ATCC 25922 was used for quality control.

### Phenotypic detection of ESBL production

ESBL production was detected using the 2011 CLSI recommendations for ESBL screening and confirmation tests [18]. The double-disc synergy test and ESBL strip E-tests (bioMerieux, Marcy L’Etoile, France) were performed for the detection and confirmation of ESBLs, respectively. For the double-disc synergy test, a ceftazidime disc (30 μg) was placed 20 mm away from a disc containing ceftazidime–clavulanate (30/10 μg). When the inhibition zone between at least one of the combination discs and its corresponding single antibiotic disc differed by ≥ 5 mm, the strain was identified as an ESBL producer. For the E-test, an ESBL strip containing ceftazidime and ceftazidime–clavulanate was used to determine the MIC ratio according to the manufacturer's instructions. *E. coli* ATCC 25922 (negative control) and *K. pneumoniae* ATCC 700603 (positive control) were used as reference strains.

### PCR of β-lactamases

Polymerase chain reaction (PCR) and sequence analyses were conducted to determine the gene responsible for the ESBL phenotype in the ESBL producers. PCR for *bla*_TEM_*, bla*_SHV_*,* and *bla*_CTX-M_ genes were conducted using PCR primers and conditions that have been previously described [19]. The five CTX-M subgroups (CTX-M-1, CTX-M-2, CTX-M-8, CTX-M-9, and CTX-M-25) were amplified using PCR primers and procedures as previously described [20].

Detection of *bla*_CTX-M-15_-like genes was performed using real-time PCR according to previously published methods [6]. Primers were used to amplify a 49-bp region that is conserved amongst group 1 CTX-M genes as follows: MC-3-15 F (5′-TGG GGG ATA AAA CCG GCA G-3′) and MC-3-15R (5′-GCG ATA TCG TTG GTG GTG C-3′). Briefly, a 25-μL reaction mixture contained final concentrations of 1× SYBR Green PCR Master Mix buffer (Applied Biosystems, CA, USA), 0.3 μM of each forward and reverse primers, 100 ng of DNA, and RNase/DNase-free water. The reaction was performed in an ABI 96-Well Optical Reaction Plate. The thermal cycling conditions were: initial denaturation at 95°C for 10 min, followed by 25 cycles at 95°C for 15 sec and 68°C for 45 sec. Following amplification, melting curve analysis was performed by heating the PCR product from 55°C to 95°C with a ramp rate of 0.05°C/s to verify the presence of a specific product according to its specific melting temperature. The results were analysed using the melting curve analysis software from Applied Biosystems.

### DNA sequencing

Automatic sequencing was performed on both strands of all PCR products using the ABI Prism 3700 DNA Sequencer (Applied Biosystems, Foster City, CA). The types of β-lactamase genes were identified by comparison with the sequences in the database of G. Jacoby and K. Bush (http://www.lahey.org/Studies/) and the sequences in GenBank (http://blast.ncbi.nlm.nih.gov/Blast.cgi).

### Pulsed-field gel electrophoresis

Pulsed-field gel electrophoresis (PFGE) of chromosomal DNA digested with *Xba*I (New England BioLabs, MA, USA) was performed according to a standard protocol [21]. Electrophoresis of the prepared samples was performed on the CHEF-DRIII system (Bio-Rad Laboratories) with 3 litres of 0.5X TBE running buffer. The gels were run with an initial switch time of 2.2 sec, a final switch time of 54.2 sec, a run time of 22 hours, an angle of 120°, a gradient of 6.0 V/cm, and a temperature of 14°C. Lambda ladder (New England BioLabs) was used as a molecular weight marker. Each gel was stained with 1 μg/ml ethidium bromide for 30 minutes and destained with distilled water for 30 minutes. The gels were photographed under UV transillumination. DNA fingerprints were analysed using BioNumerics Software (Applied Maths, Keistraat sint-Martens-laten, Belgium). Cluster analysis was performed based on the Dice coefficient with a 1.8% band tolerance and an optimisation of 4% for comparisons of DNA profiles. A similarity coefficient of 80% was applied to the generated dendrogram, which corresponds to the criteria of Tenover et al. [22].

### Detection of the ST131 clone

ST131 clone detection was performed using real-time PCR according to previously published methods [6]. The following primers were used to detect the ST131clone: ST131TF (5′-GGT GCT CCA GCA GGT G-3′) with ST131TR (5′-TGG GCG AAT GTC TGC-3′), and ST131AF (5′-GGC AAT CCA ATA TGA CCC-3′) with ST131AR (5′-ACC TGG CGA AAT TTT TCG-3′). The thermal cycling conditions for the ST131 assays included an initial denaturation at 95°C for 10 min followed by 40 cycles at 95°C for 15 sec and 60°C for 1 min. Reaction conditions and melt curve analyses were carried out as described above.

## Results

### Antimicrobial susceptibility and ESBL prevalence

Antimicrobial susceptibility testing results are shown in Table 1. Imipenem, meropenem, colistin, tigecycline, and fosfomycin were the most active agents (susceptibility: 100%). Out of the 152 strains, 50% were resistant to piperacillin/tazobactam, 51.97% to ciprofloxacin, and 41.44% to gentamicin. The resistance rates for cefotaxime, aztreonam, cefepime, and amikacin were 22.36%, 21.71%, 21.71%, and 27.63%, respectively. Over 96%, 85.5% and 71.7% of the isolates were non-susceptible to amoxicillin/clavulanic acid, piperacillin and cefoxitin, respectively.

**Table 1:** **Resistance rates for clinical *Escherichia coli***

**Antimicrobial agents**	**Number (%) of resistant isolates**		
	**ESBL (n = 31)**	**Non-ESBL (n = 121)**	**Total (n = 152)**
Amoxicillin	31 (100)	121 (100)	152 (100)
Amoxicillin/clavulanic acid	31 (100)	115 (95)	146 (96)
Piperacillin	31 (100)	99 (81.81)	130 (85.52)
Piperacillin/tazobactam	24 (77.44)	52 (42.97)	76 (50)
Cefoxitin	22(70.3)	87 (71.1)	109 (71.7)
Ceftazidime	31 (100)	2 (1.65)	33 (21.71)
Cefotaxime	31 (100)	3 (2.47)	34 (22.36)
Cefepime	31 (100)	2 (1.65)	33 (21.71)
Aztreonam	31 (100)	2 (1.65)	33 (21.71)
Imipenem	0 (0.0)	0 (0.0)	0 (0.0)
Meropenem	0 (0.0)	0 (0.0)	0 (0.0)
Ciprofloxacin	30 (96.77)	49 (40.5)	79 (51.97)
Gentamicin	26 (83.87)	37 (30.58)	63 (41.44)
Amikacin	17 (54.38)	25 (20.66)	42 (27.63)
Colistin	0 (0.0)	0 (0.0)	0 (0.0)
Tigecycline	0 (0.0)	0 (0.0)	0 (0.0)
Fosfomycin	0 (0.0)	0 (0.0)	0 (0.0)

Of the 152 *E. coli* isolates, 31 (20.4%) were confirmed to be ESBL producers. Of the 119 uropathogenic *E. coli* isolates, 11 isolates were from sputum, 10 were from stool, 9 were from blood, and three were from abscesses; 24 (20.16%), 2 (18.2%), 1 (10%), 3 (33.33%), and 1 (50%) produced ESBLs, respectively.

The susceptibility and MIC data for the ESBL-EC are summarised in Tables 1 and 2. Of all 31 isolates identified as ESBL producers, 30 isolates (96.77%) were resistant to ciprofloxacin, 17 (54.38%) to amikacin, 26 (83.87%) to gentamicin, 24 (77.44%) to piperacillin-tazobactam and 22 (70.3%) to cefoxitin. The tested strains were all susceptible to imipenem, meropenem, colistin, tigecyclin and fosfomycin, and all of them were non-susceptible to amoxicillin, amoxicillin/clavulanic acid, piperacillin, ceftazidime, cefotaxime, cefepime, and aztreonam.

**Table 2:** **Clinical specimens, MICs, PFGE, ST131 positivity, and β-lactamase genes among ESBL-producing *E. coli *** **isolates**

**No**	**Isolate**	**Specimen**	**MIC (μg/ml)**																	**Pulsotype**	**ST131**	**β-lactamases**
			**AMC**	**PIP**	**PTZ**	**CT**	**FOX**	**CAZ**	**CAZ/C**	**FP**	**IP**	**MER**	**ATM**	**GM**	**AMK**	**CIP**	**TIG**	**COL**	**FOS**			
**1**	EC22	Urine	>256	192	32	>256	2	48	32	>256	≤0.25	≤0.25	>256	>256	16	>32	≤0.25	≤0.5	≤0.25	A1	**+**	TEM-1 + CTX-M-15
**2**	EC30	Urine	>256	>256	>64	>256	0.5	64	32	>256	≤0.25	≤0.25	>256	>256	32	>32	≤0.25	≤0.5	≤0.25	A1	**+**	TEM-1 + CTX-M-15
**3**	EC11	Urine	>256	192	48	>256	0.5	48	>32	>256	≤0.25	≤0.25	>256	>256	32	>32	≤0.25	≤0.5	≤0.25	A2	**+**	TEM-1 + CTX-M-15
**4**	EC27	Urine	192	128	6	>256	16	>256	>32	>256	≤0.25	≤0.25	192	>256	6	24	≤0.25	≤0.5	≤0.25	B	**+**	TEM-1 + CTX-M-15
**5**	EC8	Urine	>256	>256	8	>256	12	>256	16	>256	≤0.25	≤0.25	>256	>256	>256	>32	≤0.25	≤0.5	≤0.25	C	**-**	TEM-1 + CTX-M-15
**6**	EC10	Urine	>256	>256	>64	>256	0.25	>256	>32	>256	≤0.25	0.5	>256	>256	6	>32	≤0.25	≤0.5	≤0.25	D	**+**	TEM-1 + CTX-M-15
**7**	EC1	Urine	>256	>256	8	>256	32	>256	16	>256	≤0.25	≤0.25	>256	>256	16	>32	≤0.25	≤0.5	≤0.25	E1	**-**	TEM-1 + CTX-M-15
**8**	EC6	Urine	>256	>256	12	>256	24	>256	8	>256	≤0.25	≤0.25	>256	>256	2	>32	≤0.25	≤0.5	≤0.25	E2	**+**	TEM-1 + CTX-M-15
**9**	EC12	Blood	>256	>256	12	>256	32	>256	8	>256	≤0.25	≤0.25	>256	64	4	4	≤0.25	≤0.5	≤0.25	E3	**+**	TEM-1 + CTX-M-15
**10**	EC9	Sputum	>256	>256	>64	>256	32	>256	8	>256	≤0.25	≤0.25	96	>256	6	>32	≤0.25	≤0.5	≤0.25	F	**-**	TEM-1 + CTX-M-15
**11**	EC31	Urine	>256	>256	>64	>256	16	>256	8	>256	≤0.25	≤0.25	>256	>256	4	2	≤0.25	≤0.5	≤0.25	G	**+**	TEM-1 + CTX-M-15
**12**	EC14	Urine	>256	>256	>64	>256	48	>256	32	>256	≤0.25	1	>256	>256	8	>32	≤0.25	≤0.5	≤0.25	H	**+**	TEM-1 + CTX-M-15
**13**	EC5	Urine	>256	>256	>64	>256	16	>256	32	>256	≤0.25	≤0.25	>256	>256	2	>32	≤0.25	≤0.5	≤0.25	I1	**+**	TEM-1 + CTX-M-15
**14**	EC32	Urine	>256	>256	>64	>256	24	128	16	>256	≤0.25	≤0.25	>256	96	2	>32	≤0.25	≤0.5	≤0.25	I2	**+**	TEM-1 + CTX-M-15
**15**	EC18	Blood	>256	>256	>64	4	32	16	2	24	≤0.25	≤0.25	48	>256	16	>32	≤0.25	≤0.5	≤0.25	J1	**-**	TEM-1 + CTX-M-15
**16**	EC20	Sputum	>256	>256	>64	>256	32	>256	>32	>256	≤0.25	≤0.25	>256	>256	48	>32	≤0.25	≤0.5	≤0.25	J2	**+**	TEM-1 + CTX-M-15
**17**	EC2	Urine	>256	>256	>64	>256	16	96	8	>256	≤0.25	≤0.25	>256	>256	4	>32	≤0.25	≤0.5	≤0.25	J3	**+**	TEM-1 + CTX-M-15
**18**	EC3	Urine	>256	>256	>64	>256	32	24	8	>256	≤0.25	≤0.25	>256	>256	8	>32	≤0.25	≤0.5	≤0.25	J4	**-**	TEM-1 + CTX-M-15
**19**	EC13	Urine	>256	>256	>64	>256	32	>256	32	>256	≤0.25	≤0.25	>256	>256	32	8	≤0.25	≤0.5	≤0.25	K1	**-**	TEM-1 + CTX-M-15
**20**	EC33	Urine	>256	>256	>64	>256	96	>256	16	>256	≤0.25	≤0.25	>256	192	64	12	≤0.25	≤0.5	≤0.25	K2	**+**	TEM-1 + CTX-M-15
**21**	EC16	Urine	>256	>256	>64	>256	24	>256	8	>256	≤0.25	≤0.25	>256	>256	64	8	≤0.25	≤0.5	≤0.25	K3	**-**	TEM-1 + CTX-M-15
**22**	EC4	Urine	>256	>256	>64	>256	0.25	192	16	>256	0.5	1.5	>256	4	2	>32	≤0.25	≤0.5	≤0.25	L1	**+**	TEM-1 + CTX-M-15
**23**	EC15	Urine	>256	>256	>64	>256	0.5	>256	18	>256	≤0.25	≤0.25	>256	4	0.5	>32	≤0.25	≤0.5	≤0.25	L1	**+**	TEM-1 + CTX-M-15
**24**	EC25	Urine	>256	>256	>64	>256	0.25	12	32	>256	≤0.25	≤0.25	>256	3	2	>32	≤0.25	≤0.5	≤0.25	L2	**-**	TEM-1 + CTX-M-15
**25**	EC26	Urine	>256	>256	>64	>256	192	>256	>32	>256	≤0.25	≤0.25	>256	>256	2	>32	≤0.25	≤0.5	≤0.25	M1	**+**	TEM-1 + CTX-M-15
**26**	EC29	Wound swab	>256	>256	>64	>256	0.25	>256	8	>256	≤0.25	≤0.25	>256	>256	6	>32	≤0.25	≤0.5	≤0.25	M2	**+**	TEM-1 + CTX-M-15
**27**	EC7	Blood	>256	>256	>64	>256	0.25	>256	8	>256	≤0.25	≤0.25	>256	2	96	1	≤0.25	≤0.5	≤0.25	N	**-**	TEM-1 + CTX-M-27
**28**	EC19	Stool	>256	>256	>64	>256	32	3	32	>256	≤0.25	≤0.25	>256	>256	128	>32	≤0.25	≤0.5	≤0.25	O	**-**	TEM-1 + CTX-M-15
**29**	EC21	Urine	128	64	8	>256	128	96	32	>256	≤0.25	≤0.25	>256	>256	>256	>32	≤0.25	≤0.5	≤0.25	P	**+**	TEM-1 + CTX-M-15
**30**	EC23	Urine	>256	192	32	>256	48	24	32	>256	≤0.25	≤0.25	>256	>256	128	>32	≤0.25	≤0.5	≤0.25	Q	**-**	TEM-1 + CTX-M-15
**31**	EC28	Urine	>256	>256	8	>256	32	4	16	>256	≤0.25	≤0.25	>256	4	2	>32	≤0.25	≤0.5	≤0.25	R	**+**	TEM-1 + CTX-M-15

*Abbreviations:AMK* Amikacin, *AMC* Amoxicillin/Clavulanic acid, *AZT* Aztreonam, *FP* Cefepime, *FOX* Cefoxitin, *CAZ* Ceftazidime, *CT* Cefotaxime, *CIP* Ciprofloxacin, *Clav* Clavulanic acid, *COL* Colistin, *GM* Gentamicin, *IP* Imipenem, *MER* Meropenem, *PIP* Piperacillin, *PTZ* Piperacillin/tazobactam, *TIG* Tigecycline, *FOS* Fosphomycin.

### β-lactamases of ESBL-producing *E. coli*

Of the 31 *E. coli* isolates with ESBL phenotypes, all tested positive for ESBL production. All ESBL types belonged to the CTX-M family; TEM- or SHV-type ESBL genes were not detected. Of the 31 ESBL-EC isolates, 30 (96.77%) produced CTX-M-15, and one (3.23%) produced CTX-M-27, a variant of CTX-M-14. Another β-lactamase, TEM-1, was concomitantly produced by 100% of all ESBL-EC isolates (Table 2).

### Pulsed-field gel electrophoresis

**Figure 1:**
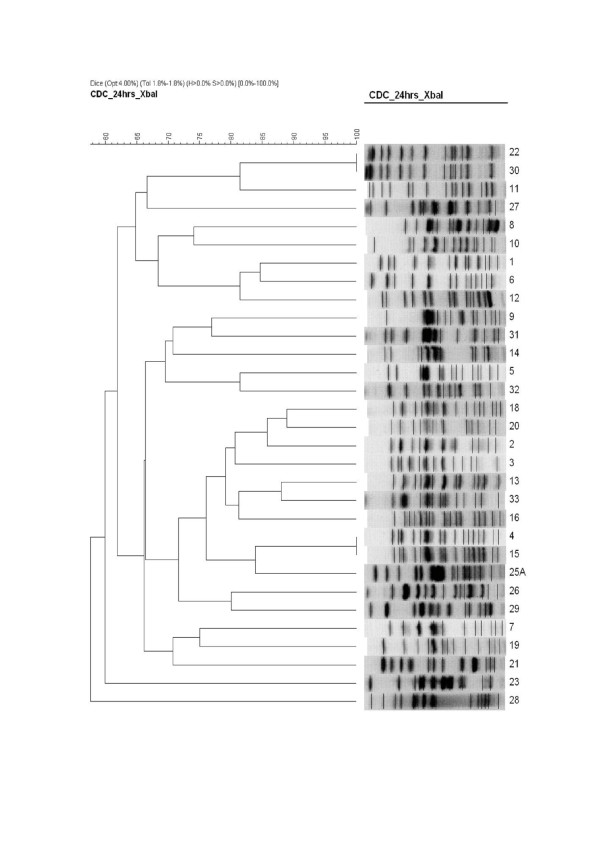
**Dendrogram of PFGE patterns showing the genetic relatedness of ESBL-producing *E. coli *** **isolates.**

PFGE was performed on 31 ESBL-EC isolates. Using a > 80% similarity cut-off point, PFGE analysis showed genetic heterogeneity in the CTX-M-producing *E. coli* isolates, revealing 29 distinctive pulsotypes (Figure 1). Twenty-seven of 29 pulsotypes comprised 87.1% of the isolates. Each pulsotype contained one strain, except for two pulsotypes, A1 and L1, which contained two strains each. Two pulsotypes represented 12.9% of all studied ESBL-EC isolates. The results of PFGE revealed that there were no outbreaks or spreading of one single pulsotype in the hospital.

### Prevalence of ST131 in ESBL-EC

Twenty (64.5%) of 31 ESBL-EC isolates were identified as the ST131 clone. The ST131 ESBL-ES isolates belonged to 18 different pulsotypes. All ST131 isolates produced CTX-M-15. Sixteen (66.6) of 24 uropathogenic ESBL-EC isolates were ST131, while two (66.6%) of three blood isolates were ST131, one (50%) of two ESBL-EC isolated from sputum was ST131, and one (100%) ESBL-EC isolated from a wound was ST131.

## Discussion

The prevalence of ESBLs among clinical isolates, especially *E. coli* and *K. pneumoniae*, has increased significantly over the past two decades [3]. Despite this increase in ESBLs worldwide, there is a paucity of local reports on the prevalence of ESBL-EC [14-16]. Bindayna et al. [17] reported a high level of *bla*_CTX-M_-positive ESBL-EC; however, they did not determine the type of CTX-M enzymes present. This study is the first in Saudi Arabia to specifically determine the CTX-M-types of ESBLs, to determine strain typing and to determine the spread of the ST131 clone among ESBL-EC isolates sampled from inpatients.

A total of 152 *E. coli* clinical isolates collected from a hospitalised patient population over a 10-month time period from September 2010 to June 2011 were evaluated for the production of ESBL enzymes. The results revealed that 31 (20.4%) of 152 *E. coli* isolates produced ESBL. This study demonstrated an increasing prevalence of ESBL-EC. In previous local studies, the prevalence rates of ESBL-EC were 6.5% and 10.3% in 2002 and 2004, respectively [14], and the prevalence rates were 15.7% and 4.8% from inpatients and outpatients, respectively [15]. Recently, the prevalence of ESBL-EC in uropathogenic *E. coli* was 33.3% [16].

Currently, CTX-M enzymes are replacing SHV and TEM enzymes as the prevalent ESBL type [2]. In this study, all ESBL-EC produced the ESBL CTX-M concomitant with the narrow-spectrum β-lactamase TEM-1. None of the isolates produced SHV β-lactamase. From our study, we documented that CTX-M enzymes are the dominant ESBLs in Saudi Arabia. CTX-M-15-producing *E. coli* have spread worldwide [1]. Our results revealed that CTX-M-type enzymes accounted for 100% of all ESBLs. These results reflect the global trend toward a pandemic spread of CTX-M-type ESBLs in *E. coli*. While the CTX-M-1 group was predominant, the CTX-M-9 group was rarely reported in Saudi Arabia: within the CTX-M family, CTX-M-1- and CTX-M-9-like genes had prevalence rates of 96.77% and 3.23%, respectively. DNA sequencing revealed that CTX-M-15 is the sole enzyme with a CTX-M-1-like gene, while CTX-M-27, a variant of CTX-M-14, is the sole enzyme with a CTX-M-9-like gene. This is the first study to describe CTX-M-15 and CTX-M-27 enzymes among ESBL-EC isolates in Saudi Arabia and to document a high prevalence of CTX-M-15. In previous local studies, two groups of enzymes; CTX-M-1- and CTXM-9-like genes, were identified in CTX-M-positive *K. pneumoniae* isolates, with prevalence rates of 60% and 40%, respectively [10]. CTX-M-15 and CTX-M-27 enzymes hydrolyse cefotaxime and ceftazidime. CTX-M-27 confers stronger resistance to ceftazidime than CTX-M-14 [23]. Strikingly, we identified one ESBL-EC isolate producing CTX-M-27 in Saudi Arabia for the first time.

*E. coli* ST131 is a worldwide pandemic clone that causes antimicrobial-resistant infections. The pandemic spread of CTX-M-15 ESBL-EC was identified in 2008 on three continents [7,24]. Since then, the worldwide prevalence of ST131 harbouring a broad range of virulence and resistance genes on a transferable plasmid has been confirmed [7]. *E. coli* ST131 can harbour a variety of β-lactamase genes; most often, these include CTX-M, and, less frequently, TEM, SHV, and CMY. The *E. coli* ST131 clone has been reported worldwide; this clone is frequently multidrug-resistant, commonly carries CTX-M-15 [6,8]. CTX-M-15-producing *E. coli* ST131 has not yet been reported in Saudi Arabia. A particularly interesting result of this study is the finding that ST131 was prevalent among *bla*_CTX-M-15_-positive *E. coli* isolates (20/30; 66.66%) in Saudi Arabia. However, the CTX-M-27-positive isolates were not ST131. Our results suggested that ST131-CTX-M-15-ESBL-EC strains have circulated in Saudi Arabia. A high prevalence of the clone (∼30%–60%) has been identified amongst fluoroquinolone-resistant *E. coli*. ST131 comprised 64% of community-acquired and 84% of hospital-acquired cefpodoxime-resistant *E. coli* infections [25]. ST131 comprised 53% and 30% of CTX-M ESBL-producing *E. coli* in Chicago and Pittsburgh, respectively [26,27]. In Indonesia, 36.8% of CTX-M-15-positive *E. coli* belonged to the O25b-ST131 lineage [28]. A high prevalence of the CTX-M-15-producing O25b-ST131 *E. coli* clone in Bulgaria has been reported [29], as have high proportions of ESBL-producing *E. coli* ST131 isolates from India, Pakistan, Iran, and Lebanon [30]. In early studies, ST131 clones producing CTX-M-15 had the propensity to cause community-onset infections, especially urinary tract infections [30-32]. However, this clone has also been recently identified in isolates recovered from health care settings, such as hospitals and nursing homes [33,34]. There was a high proportion of ST131 among CTX-M-15 producing isolates.

Limitations of this study include that it was performed at a single institution in Saudi Arabia and that a low number of isolates were studied. Furthermore, this study was conducted on hospitalised patients, which does not reflect the epidemiological changes in the Saudi community. Thus, the study results may not reflect the epidemiology of different geographic areas within the kingdom. Despite these limitations, to our knowledge, this is the first study describing the genetic characteristics of ESBL-EC in Saudi Arabia. This study demonstrates a much higher overall prevalence of ESBL-EC than has been previously reported in Saudi Arabia, with CTX-M-type enzymes being the predominant ESBLs. In addition, the study results provide evidence for the dominance of CTX-M-15-producing isolates in Saudi Arabia [9,10,12,13,17] and that the predominance of ST131 is likely responsible for the clonal expansion of ESBL-EC in Saudi Arabia, conclusions that are consistent with the changing epidemiology of ESBL-EC worldwide.

## Competing interests

The authors declare that they have no competing interests.

## Authors’ contributions

MHA conceived and designed the study, carried out the antibiotic sensitivity, phenotypic detection of ESBL, molecular genetic studies, participated in the sequence alignment and drafted the manuscript. AMS carried out the PFGE and participated in phenotypic detection of ESBL and drafted manuscript. MMH carried out the real-time PCR for detection of ST131. MNA participated in the PFGE. ZAM participated in the design of the study. HK collected the isolates and the clinical data. All authors read and approved the final manuscript.
